# Effects of Vegetarian Nutrition–A Nutrition Ecological Perspective

**DOI:** 10.3390/nu2050496

**Published:** 2010-05-10

**Authors:** Martina Metz, Ingrid Hoffmann

**Affiliations:** 1 Institute of Nutritional Science, Justus Liebig University Giessen, Wilhelmstrasse 20, D-35392 Giessen, Germany; Email: Martina.Metz@ernaehrung.uni-giessen.de; 2 Max Rubner-Institut, Federal Research Institute of Nutrition and Food, Department of Nutritional Behaviour, Haid-und-Neu-Straße 9, D-76131 Karlsruhe, Germany

**Keywords:** vegetarian nutrition, nutrition ecology, interrelatedness, cause-effect chains

## Abstract

Although vegetarian nutrition is a complex issue, the multidimensionality and interrelatedness of its effects are rarely explored. This article aims to demonstrate the complexity of vegetarian nutrition by means of the nutrition ecological modeling technique NutriMod. The integrative qualitative cause-effect model, which is based on scientific literature, provides a comprehensive picture of vegetarian nutrition. The nutrition ecological perspective offers a basis for the assessment of the effects of worldwide developments concerning shifts in diets and the effects of vegetarian nutrition on global problems like climate change. Furthermore, new research areas on the complexity of vegetarian nutrition can be identified.

## 1. Introduction

“We must recognize complexity before we can deal with it” [1]

Vegetarian nutrition is a complex issue. Scientific statements regarding the effects of vegetarian nutrition need to take into account the interrelatedness of a large number of effects that are subject to mutual influences and constant changes [2,3,4,5]. The aim of this article is to demonstrate the complexity of vegetarian nutrition by modeling the interrelatedness of its effects, and consequently, to demonstrate the implication for statements on vegetarian nutrition.

The article is based on a new concept dealing with complexity and multidimensionality in the field of nutrition: Nutrition ecology is a relatively new scientific approach and deals with complex nutrition-related problems or issues like vegetarian nutrition.

### The nutrition ecological approach

**Figure 1:**
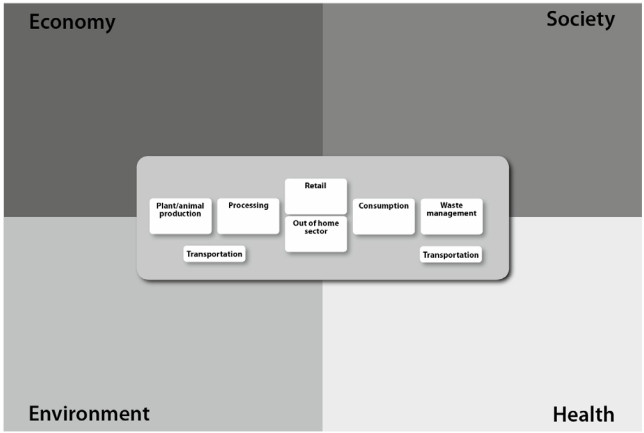
The nutrition ecological approach - the four dimensions of nutrition and the complete food supply chain are taken into account.

Since the term ‘nutrition ecology’ is used to refer to the complex interactions among components or aspects of nutritional issues, it is not limited to the association of nutrition and environment (econutrition). Instead the concept covers four dimensions of nutrition: health, environment, society and economy. The dimensions are taken into account simultaneously and equally [6]. In addition, all parts of the food supply chain are considered - from agricultural production via processing, retail and the out of home sector to consumption and waste management, with transportation between each step (Figure 1). Applying the concept of nutrition ecology to vegetarian nutrition allows including the complete spectrum of vegetarian nutrition.

By means of the modeling technique NutriMod the complexity and multidimensionality of vegetarian nutrition is captured to support problem-solving processes [6,7].

## 2. The Model of Vegetarian Nutrition

**Figure 2:**
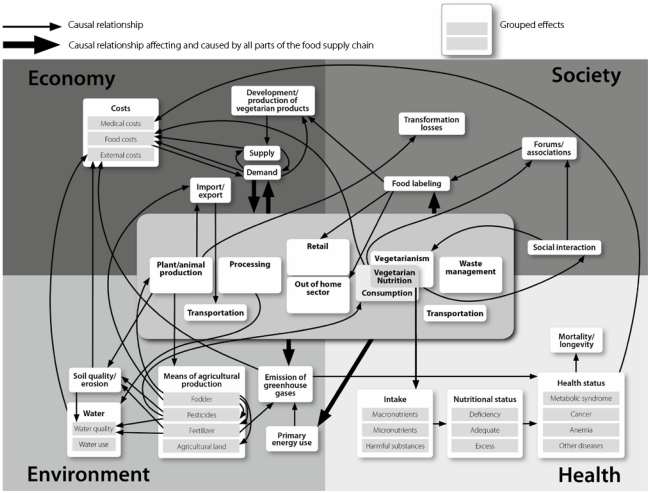
Qualitative model of the effects of vegetarian nutrition.

An integrative qualitative model (Figure 2) was developed with the modeling technique NutriMod by compiling the effects of vegetarian nutrition as described in the scientific literature for the four dimensions of nutrition. 

By means of scientific literature research, the effects of lacto-, ovo-, lacto-ovo-vegetarian and vegan diets in industrialized countries were collected and the studied relationships documented. Results of the applied studies are documented in tables including a description of the relationship and the relevant literature. In the model, this information lies behind each arrow. An electronic version of the model with hypertext structure containing examples of references is available online: http://www.uni-giessen.de/fbr09/nutr-ecol/forsc_veg_e.php [8].

The model integrates the results of 150 studies. These were identified by literature search in several thematically differing scientific databases according to the four dimensions of nutrition e.g., Agris, Agricola, EconLit, MEDLINE, Sociological Abstracts, SciSearch and Web of Science between October 2007 and July 2009. A variety of keywords was used, e.g., for the dimension environment: vegetarian and water use, pesticides, fertilizer, greenhouse, energy use, land use, agricultural land, pollution, livestock, *etc*.; for the dimension health: vegetarian or vegan and anemia, macronutrients, micronutrients and keywords for diverse diseases.

The aspects described in the scientific literature were transferred into the model as ‘components’. These terms are written in italic. 

The model is limited to the effects of vegetarian nutrition, which represents the intersection of vegetarianism and the nutrition of the general population, in the model called ‘consumption’ (Figure 2). Vegetarianism encompasses an overall vegetarian lifestyle including health consciousness or avoiding animal products also in non-food items (e.g., certain cosmetics), which is not part of the model. Consequently, aspects like education and income as part of vegetarianism, but not of vegetarian nutrition, are also not included in the model.

The nutrition ecological approach as described above (introduction) was applied to examine the effects of vegetarian nutrition in the four dimensions of nutrition along the food supply chain. Therefore, in the qualitative model, each aspect described in the literature was explicitly allocated to one of the four dimensions, even though some effects of vegetarian nutrition might be allocated to more than one dimension. For example, vegetarian nutrition is associated with a lower energy use along the food supply chain. This is an aspect of the dimension environment when considering the lower emissions of carbon-dioxide (CO_2_) equivalents and its association with global warming. It is an aspect of the dimension society when the distribution of primary energy within and across generations is considered as a question of justice. Furthermore, examining its costs, energy use belongs to the dimension economy. In the model, energy use is explicitly allocated to the dimension environment since this aspect is usually described in the scientific literature [9].

The model visualizes the multitude of effects of vegetarian nutrition. Each component in the model summarizes different aspects of vegetarian nutrition, e.g., ‘*micronutrients’* summarizes vitamins, minerals, and phytochemicals. Some aspects are aggregated under topics, such as ‘*intake’* for the intake of *macronutrients*, *micronutrients* and *harmful substances*. 

Each of the dimensions comprises components, which are interrelated both within and across the dimensions [6]. Causal relationships between two components are represented by arrows. This results in cause-effect chains and demonstrates the interrelatedness of the effects of vegetarian nutrition (Figure 2).

The present investigation on the interrelated effects of vegetarian nutrition reaches beyond the mostly detail oriented scientific work. Compiling the results of the scientific literature into an integrated qualitative model of vegetarian nutrition portrays the complexity of this issue.

The literature search underlines that research on vegetarian nutrition has focused mainly on health effects and, more recently, increasingly on environmental aspects. Little is known about its societal and economic effects and virtually nothing about the interrelatedness of the various effects. 

Even though it is a static model in which dynamics are not directly visible, it promotes awareness of dynamics in time and space by depicting the interrelatedness. An example for dynamics in time is the *health status* of the general population that may only be affected by vegetarian nutrition with a time delay. Other effects of vegetarian nutrition may occur only within a century, meaning that a person may not experience the effect which he or she intended, e.g., curbing climate change through lower *emissions of greenhouse gases*. Dynamics in space means that the effect happens at another place than the releasing cause. An example is that vegetarian nutrition practiced in an affluent country may affect via many intermediate steps food security in countries of scarcity. Vegetarian nutrition may reduce the worldwide *animal production* and the *transformation losses* as well as the demand for fodder and consequently increase the availability of agricultural land for *plant production*. 

Currently, two developments can be observed worldwide that require a comprehensive assessment of the actual effects of vegetarian nutrition. Firstly, a relevant proportion of the world’s population shifts towards a more animal-based diet. In economically quickly developing countries like India and China a decreasing number of persons practice plant-based diets [10]. In affluent countries, both an increase and a decrease of plant-based diets can be observed. Secondly, there is increasing global problems that reinforce one another. For example, world hunger is increased by the consequences of climate change such as droughts and crop shortages [11], by the growing world population and the rising worldwide demand for food. Even though the presented model features the (re)integration of information and, therefore, reflects the limitations of the available data, it provides the basis for an assessment of the effects of shifts in diets worldwide and of the effects of vegetarian nutrition on global problems. Via many intermediate steps, vegetarian nutrition mostly attenuates global problems such as climate change, shortage of natural resources, world hunger, poverty, and chronic diseases e.g., by a decrease in livestock production, a reduced demand for fodder and the avoidance of the conversion of animal food to human food [9,12,13]. In this sense, vegetarian nutrition may be a means of co-responsibility for other humans and the global living environment [14].

## 3. Insights into Vegetarian Nutrition from A Nutrition Ecological Perspective

Applying the nutrition ecological approach allows insights into vegetarian nutrition that are not apparent when limiting the perspective to single aspects or one-dimensional research.

**Figure 3:**
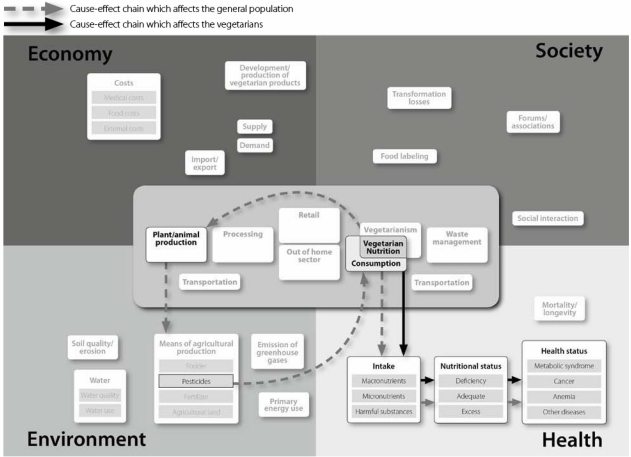
Example for the effects of vegetarian nutrition, which happen in parallel and may affect vegetarians as well as the general population.

The developed model portrays that the many different effects of vegetarian nutrition happen in parallel. For example, as shown in Figure 3, practicing vegetarian nutrition for personal benefits has effects on the vegetarians’ health status (e.g., as summarized in [15,16,17]). At the same time, lower agricultural production of foods of animal origin is associated with less use of pesticides [9], which results in lower concentrations of residues in water and soil. This means a lower intake of these residues by the general population (including vegetarians). It indicates that vegetarian nutrition affects the vegetarians themselves as well as the general population.

Some effects of vegetarian nutrition are related to the higher consumption of plant-foods by vegetarians, e.g., health effects [15,16,17], and others are due to omitting foods of animal origin, especially environmental effects.

Vegetarian nutrition has direct and indirect effects. For example, it directly affects the *intake* of *macro- and micronutrients* and *food costs* [17,18,19,20] (Figure 3). Proceeding from direct effects, the model reveals indirect effects of vegetarian nutrition. For example, *health status* may be perceived as a direct effect of vegetarian nutrition, but is actually an indirect effect because it results from the *nutritional status*, which in turn derives from the specific *intake* when practicing *vegetarian nutrition* (Figure 3).

Vegetarian nutrition causes changes in all parts of the food supply chain. Omitting food of animal origin affects *retail* and *out of home sector*, which in turn affects *processing*, *plant & animal production*, and finally *waste management* as well as *transportation*. Each shift in one part of the food supply chain results in further effects leading to cause-effect chains. An example of a cause-effect chain is the association of *plant production* with *energy use*, *CO_2_ emissions* and finally *health effects. Plant production* is associated with lower *energy use* compared to *animal production* with its high energy use for the production of fodder. A lower *energy-use* results in lower *CO_2_ emissions* [9], which curbs climate change [13]. As climate may affect health, e.g., concerning skin diseases or allergies, curbing climate change is ultimately associated with beneficial health effects of vegetarian diets [21] which in turn may lower medical costs. Furthermore, this cause-effect chain is an example of the multidimensionality of vegetarian nutrition since there are effects within and across the dimensions of nutrition.

The compilation of the effects of vegetarian nutrition depicts intended effects (e.g., association with *health status*) as well as unintended effects or side-effects. Potential negative unintended effects might be the loss of employment for those working in animal production or less employment in the health sector because of a more favorable health status of vegetarians. A positive unintended effect could be that new opportunities may arise like jobs for the *development* and *production of vegetarian products*. 

## 4. Discussion

The presented compilation provides insights into a more comprehensive picture of vegetarian nutrition: vegetarian nutrition has both short and long-term effects on all four dimensions of nutrition and along the food supply chain; it has effects at an individual and population level, on a local and global scale; and affects vegetarians as well as the general population, at present and in the future. Even if vegetarians practice their diet just for their personal benefit, all effects happen in parallel. Depicting how the effects of vegetarian nutrition are interrelated reveals cause-effect chains, intended and unintended effects, both positive and negative, as well as dynamics in time and space.

Investigating single aspects or the combination of only a few aspects may lead to a formally accurate scientific assessment, but still provides a very restricted and biased view of reality [5]. Therefore, for a comprehensive understanding of this complex issue, all relevant aspects need to be captured in their interrelatedness. Compared with the limited knowledge available a more realistic and profound assessment of the actual effects of vegetarian nutrition is necessary to answer questions like the one about the impact of a higher life expectancy of vegetarians [22] on medical insurance.

For the validation of the qualitative model further research is required. Studies on long-term effects, on cause-effect chains within and across the dimensions, on the dynamics of effects, on effects on a global scale, on future generations, and on societal and economic aspects are necessary. This would provide the basis for quantifying and simulating the effects of vegetarian nutrition. 

Besides research interests, the model based on the nutrition ecological approach may support actors in practice along the food supply chain in decision-making e.g., for the development of new products. The gained insights may also serve as a basis for decision-making in politics, counselling and individual dietary choices. The model gives orientation concerning the complexity of vegetarian nutrition and may bring additional aspects into focus.
